# Pericardial Tamponade After Systemic Alteplase in Stroke and Emergent Reversal With Tranexamic Acid

**DOI:** 10.5811/cpcem.2019.10.44369

**Published:** 2019-12-17

**Authors:** Cynthia Romero, Samuel Shartar, Michael J. Carr

**Affiliations:** * Emory University Hospital, Department of Emergency Medicine, Atlanta, Georgia; † Emory University Hospital, Office of Critical Event Preparedness and Response, Atlanta, Georgia

## Abstract

Alteplase, or tissue plasminogen activator (tPA), lyses clots by enhancing activation of plasminogen to plasmin. Conversely, tranexamic acid (TXA) functions by inhibiting the conversion of plasminogen to plasmin, which inhibits fibrinolysis. TXA has proven safe and effective in major bleeding with various etiologies. A 76-year-old male developed acute ischemic stroke symptoms. Systemic alteplase was administered and he showed clinical improvement. Shortly thereafter, the patient became hypotensive and lost pulses. Point-of-care ultrasound revealed cardiac tamponade. TXA was immediately given to inhibit fibrinolysis since cryoprecipitate and blood products were not immediately available. Pericardiocentesis was performed and successfully removed 200 milliliters of blood with return of pulses. Clinicians must consider TXA as a rapidly accessible antagonist of tPA’s fibrinolytic effects.

## INTRODUCTION

The current treatment for acute ischemic stroke is to restore blood flow to the brain using reperfusion therapy. Several options have proven effective in reperfusion therapy including systemic or local tissue plasminogen activator (tPA or alteplase), or mechanical thrombectomy.^1^ Thrombolytic therapy with tPA is considered the mainstay of treatment of acute ischemic stroke. Alteplase acts as a thrombolytic by binding to fibrin in the thrombus and enhancing the activation of plasminogen to plasmin. Plasmin lyses the thrombus into fibrin degradation products, thus initiating local fibrinolysis.^1^

In the United States, approximately 795,000 strokes occur each year, of which 692,000 are ischemic strokes.^2^ Of these, it is estimated that 3.4%–5.2% of ischemic stroke patients receive tPA, which is between 24,000 and 36,000 stroke patients yearly.^1^ Thrombolytics have many risks, of which the most feared complication is intracranial hemorrhage or major bleeding elsewhere in the body.^3^ Pericardial tamponade is a rare adverse effect of tPA with only a few cases reported, usually associated with recent myocardial infarction.^3^–^5^

Tranexamic acid (TXA) has been safely used as an antifibrinolytic in a full spectrum of clinical settings. Its on- and off-label use has rapidly expanded over the past few decades.^6^ TXA works by blocking the conversion of plasminogen to plasmin and prevents plasmin from binding to fibrin, inhibiting clot breakdown and abating hemorrhage.^7^ There is no data regarding the efficacy or safety of the routine use of TXA to reverse tPA-induced fibrinolysis. Given its molecular mechanism of action, however, TXA is the perfect antagonist to tPA and should be considered in cases of life-threatening hemorrhage secondary to tPA administration.

## CASE PRESENTATION

A 76-year-old male with past medical history of hypertension presented to the emergency department (ED) by ambulance as a stroke alert. History was obtained from the patient’s family and emergency medical services (EMS). The patient was in his usual state of health, speaking to his daughter on the phone, when he abruptly stopped talking and was no longer responding to her. This prompted her to call 9-1-1. On EMS arrival, the patient appeared to be convulsing, which quickly resolved, and he was then nonverbal but remained interactive. He was noted to have left-sided hemiparesis and was expressively aphasic. The patient was not on anticoagulants and family denied any recent trauma, bleeding, or surgery.

Initial physical examination revealed a nonverbal, elderly gentleman in mild distress. He was aphasic. Vital signs were as follows: blood pressure 98/62 millimeters of mercury (mmHg), pulse 76 beats per minute, respirations 19 breaths per minute, oral temperature 36 degree Celsius (°C) (96.8°F), and pulse oximetry 99% on room air. Neurologic examination was notable for pupils equally round and reactive to light, left visual field loss with right gaze preference, left facial droop, and localization of painful stimuli on the right with absence of painful withdrawal on the left upper and lower extremities. He demonstrated left upper and lower extremity hemiparesis. National Institutes of Health Stroke Scale was 18. Pulmonary examination revealed lungs clear to auscultation, and cardiac examination revealed a regular rate and rhythm with no murmurs, gallops, or rubs. There was normal peripheral perfusion in all extremities.

Labs were notable for point-of-care (POC) glucose 114 milligrams per deciliter (mg/dL) (reference range: 65 – 110 mg/dL), POC creatinine 0.66 mg/dL (reference range: 0.8 – 1.3 mg/dL), POC international normalized ratio (INR) 1.0 (reference range: 0.9 – 1.2), troponin-I < 0.03 nanograms per milliliter (ng/mL) (reference range: 0 – 0.4 ng/mL) and complete blood count notable for platelet count 143 × 10^9^ per liter (L) (reference range: 150 – 400 × 10^9^/L). Non-contrasted computed tomography (CT) of the head was grossly within normal limits. CT angiogram (CTA) of the head and neck did not demonstrate dissection or a large vessel occlusion, and the patient was not a candidate for thrombectomy. Evaluation of the aorta was markedly suboptimal, with mild aortic wall thickening. The heart was not visualized in the CTA head and neck study. As the patient was within the treatment window, tPA was administered one hour after symptom onset for his right middle cerebral artery syndrome acute ischemic stroke.

On re-examination 20 minutes after tPA administration, the patient was returning to his mental baseline with improving neurologic exam. He was noted to be awake, alert, and talking to his family. Approximately 45 minutes after tPA administration, the patient had an acute change in mental status, acute onset vomiting, and was noted to be hypotensive to 61/46 mmHg. Focused assessment with sonography in trauma (FAST) exam using point-of-care ultrasound revealed a large pericardial effusion with tamponade physiology, specifically right ventricular diastolic collapse (Image). He then lost pulses and cardiopulmonary resuscitation (CPR) was initiated. TXA was immediately given to inhibit tPA-induced fibrinolysis and bleeding.

Compressions were halted and pericardiocentesis was performed with 200mL of dark blood removed using a triple lumen catheter (TLC) kit. An ultrasound-guided approach with Seldinger technique was used to insert the TLC into the pericardial space. The patient subsequently regained strong pulses and began to wake up and respond to commands. Two units of uncrossed blood were then given to him. Cryoprecipitate was ordered but not immediately available. His blood pressure recovered to 179/117 mmHg. No epinephrine or other Advanced Cardiovascular Life Support medications were given during the period of cardiac arrest. Repeat point-of-care cardiac ultrasound showed resolution of tamponade physiology.

**CPC-EM Capsule**

What do we already know about this clinical entity?

Alteplase (tPA) causes lysis of fibrin clots by enhancing the activation of plasminogen to plasmin. A feared adverse effect of this medication is major hemorrhage.

What makes this presentation of disease reportable?

We report a rare, adverse effect of tPA administration causing cardiac tamponade and profound hypoperfusion, temporarily abated with tranexamic acid (TXA) and pericardiocentesis.

What is the major learning point?

TXA should be considered as an adjunct therapy for ongoing hemorrhage. Its molecular mechanism of action makes it the ideal antagonist to tPA’s fibrinolytic effects.

How might this improve emergency medicine practice?

It is imperative to evaluate patients who become hypotensive after tPA with point-of-care ultrasound, including echocardiography. Consider TXA to abate hemorrhage.

Cardiothoracic surgery was consulted, and they exchanged the TLC catheter for a pericardial drain over a wire in the ED. No additional blood was noted to drain from the new pericardial drain. The patient was admitted to the intensive care unit (ICU) and was awaiting transport out of the ED. During this time his family was bedside, talking with the patient. Over the next hour, he was treated with 10 units of cryoprecipitate. Approximately 3.5 hours after arrival to the ED, the patient again became unresponsive and was noted to be hypotensive and bradycardic. Point-of-care ultrasound showed re-accumulation of pericardial effusion and worsening tamponade physiology.

Unfortunately, the existing pericardial drain was not functioning even after flushing the catheter. The patient again lost pulses and CPR was initiated. A second pericardiocentesis was performed by cardiothoracic surgery with removal of 800 mL of bright red blood. Despite removing the blood, the tamponade was not resolving as the re-accumulation was too great. After discussion with the patient’s family, care was withdrawn and CPR discontinued, in line with their wishes. The patient expired shortly after termination of resuscitative efforts.

## DISCUSSION

The treatment of acute ischemic stroke focuses on tPA administration to improve neurologic outcomes. This treatment has many well-documented complications, including intracerebral hemorrhage, systemic bleeding, angioedema, spontaneous hemothorax, and spontaneous pericardial tamponade in the setting of recent myocardial infarction.^3^,^4^,^8^ The incidence of fatal hemopericardium after tPA administration in stroke treatment is unknown.

Currently there are no approved alternatives to blood products for the reversal of tPA-induced fibrinolysis.^9^ Treatment options for intracranial hemorrhage related to tPA administration are unproven, but include platelets, cryoprecipitate, and consideration of prothrombin complex concentrate and fresh frozen plasma.^10^ As our patient’s condition was life threatening and there was not enough time to wait for blood products to be prepared, he was immediately treated with TXA and pericardiocentesis. There was no re-accumulation of blood in the pericardial space on repeat point-of-care ultrasound. This is the first case, to our knowledge, of cardiac tamponade with cardiac arrest after systemic tPA administration for acute ischemic stroke successfully temporized with TXA, in addition to emergent pericardiocentesis. Even though the patient ultimately died, the TXA seemed to halt the re-accumulation of blood, giving the patient time to wake up and speak to his family, which is invaluable for critically ill patients with life-threatening injuries.

TXA is an antifibrinolytic and a lysine analog that occupies binding sites on plasminogen, thus preventing its binding to fibrin and inhibiting plasminogen activation to plasmin. The blockade of lysine-binding sites on plasmin prevents binding to fibrin and thus inhibits clot breakdown.^6^ TXA causes a delay in the body’s normal physiologic breakdown of platelet aggregation and ensures existing clots remain viable, lessening hemorrhage.^7^ Given its mechanism of action at the molecular level, TXA is a logical antidote for reversing the thrombolytic effects of tPA.

Most major studies of TXA use have been in the setting of traumatic hemorrhage. The largest study of TXA is the Clinical Randomization of an Antifibrinolytic in Significant Hemorrhage 2 (CRASH-2) trial, which revealed that TXA decreased the risk of death in bleeding trauma patients.^11^ Literature review of TXA’s safety and efficacy shows its wide applicability outside of the trauma setting. It has been safely used in coagulopathies, to control heavy menstrual cycle bleeding, to reduce death due to bleeding in post-partum hemorrhage, perioperatively to reduce bleeding in adult patients having elective posterior thoracic/lumbar spinal fusion surgery, and in patients undergoing cardiac surgery.^6^,^7^,^12^–^14^ Numerous studies and Cochran Review concluded that TXA has a good safety profile when administered within three hours of injury and demonstrated no evidence that TXA has an effect on the risk of vascular occlusive events.^7^,^11^,^15^,^16^

Based on all available multidisciplinary data, TXA appears safe when used in the right timeframe without increasing risk of thrombotic events. While traditionally used in the setting of traumatic hemorrhage, it should be considered to counteract acute hemorrhage secondary to tPA administration. Again, at the molecular level, it directly counteracts the mechanism of action of tPA and can therefore be lifesaving or temporize life-threatening injuries while definitive treatment is provided. In the case of our patient, it is unclear what led to the spontaneous hemopericardium. His initial aphasia secondary to acute stroke complicated our ability to obtain a full history in order to determine if he had any recent symptoms suggesting myocardial infarction. There was also no cardiac imaging completed prior to the initial point-of-care ultrasound during the patient’s resuscitation. It is not known whether there was a pre-existing pericardial effusion. Clinically, we must also consider the possibility that the stroke and cardiac tamponade were due to an aortic dissection. No autopsy was performed to confirm this. Regardless of the exact etiology of the pericardial tamponade, it is feasible that patients with hemopericardium after tPA administration may benefit from TXA.

## CONCLUSION

This case report describes the occurrence of a rare adverse effect of tPA administration, bleeding into the pericardium causing cardiac tamponade and profound hypoperfusion, temporarily abated with TXA and pericardiocentesis. This rare complication may be missed if clinicians do not maintain a high index of suspicion. Pericardiocentesis is the definitive treatment for acute pericardial tamponade. While this will serve to evacuate the existing pericardial effusion, it will not stop any further bleeding into the pericardial space and, thus, TXA should be considered as an adjunct therapy to impede ongoing hemorrhage. While traditionally used in the setting of traumatic hemorrhage, its molecular mechanism of action makes it the ideal antagonist to tPA’s fibrinolytic effects. The bottom line is to consider TXA as soon as possible in the setting of massive or life-threatening hemorrhage secondary to tPA thrombolysis.

## Figures and Tables

**Image:**
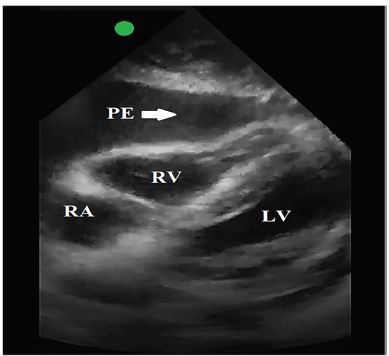
In this subxiphoid view obtained with a phased array, point-of-care ultrasound probe, there is obvious pericardial effusion with right ventricular wall collapse. This demonstrates pericardial tamponade physiology in combination with the patient’s vital signs and physical exam findings. *RV*, right ventricle; *LV*, left ventricle; *RA*, right atrium; *PE*, pericardial effusion.
